# Neuropsychiatric Symptoms as Indicators of Fall Risk in Geriatric Inpatients

**DOI:** 10.3390/medicina59050887

**Published:** 2023-05-05

**Authors:** Krzysztof Wilczyński, Marta Gorczyca, Małgorzata Grabarczyk, Jan Szewieczek

**Affiliations:** 1 Department of Geriatrics, Faculty of Health Sciences in Katowice, Medical University of Silesia in Katowice, Ziołowa 45/47, 40-635 Katowice, Poland; 2 Department of Anatomy, Institute of Medicine, University of Opole, Oleska 48, 45-052 Opole, Poland; 3 Faculty of Medical Sciences, University of Applied Sciences, Ujejskiego 12, 48-300 Nysa, Poland; 4 Health Promotion and Obesity Management Unit, Department of Pathophysiology, Faculty of Medicine in Katowice, Medical University of Silesia in Katowice, Medyków 18, 40-752 Katowice, Poland

**Keywords:** elderly, dementia, cognitive impairment, falls, risk of falls, neuropsychiatric symptoms

## Abstract

*Background and Objectives*: It is well established that patients with cognitive impairment are at a higher risk of falls. However, the impact of coexisting neuropsychiatric symptoms on the overall risk of falls in hospitalized geriatric individuals with and without dementia has not been extensively studied. This cross-sectional study will assess the association between neuropsychiatric symptoms and fall risk in geriatric individuals analyzed by sex. *Materials and Methods*: A total of 234 patients, both with and without dementia, admitted to the geriatric ward at Leszek Giec Upper-Silesian Medical Centre of the Silesian Medical University in Katowice, Poland, between January 2019 and January 2020 were included in this study. The Neuropsychiatric Inventory–Questionnaire was used to assess the presence of neuropsychiatric symptoms. Increased fall risk was defined by Berg scores of ≤40. *Results*: The mean age of the study group was 80.7 ± 6.6, and women accounted for 62.8% of the study population. Apathy was the most common neuropsychiatric symptom, affecting 58.1% of patients, and it was the most common symptom among people with dementia, affecting 67.80% of patients. The receiver operating characteristics curve analysis revealed that a high fall risk was significantly associated with the total number of neuropsychiatric symptoms (≥4) and the total intensity of these symptoms (≥6). For women, high fall risk was associated with three or more neuropsychiatric symptoms and a total neuropsychiatric symptom intensity score of at least 6. For men, the association of high fall risk with the total number of NPS was not significant; a total NPS intensity score of 10 or more was associated with high fall risk. Multivariate logistic regression analysis identified associations with fall risk for hallucinations. *Conclusions*: Our results suggest that the presence of neuropsychiatric symptoms, particularly hallucinations is associated with an increased risk of falls in geriatric inpatients. In addition, the cumulative total of NPS and their cumulative intensity are both independently associated with an increased risk of falls. These results suggest that fall prevention strategies should include the management of neuropsychiatric symptoms in hospitalized geriatric individuals.

## 1. Introduction

Neuropsychiatric symptoms (NPS) are often associated with dementia. It is estimated that up to 97% of individuals with dementia will experience NPS, including psychiatric symptoms such as apathy, irritability, anxiety, dysphoria, aggression, delusions, and hallucinations, as well as behavioral disorders such as nighttime disturbances, aberrant motor activity, and disinhibition [1,2]. However, not only persons with dementia may manifest NPS [3]. The presence of NPS can have a significant impact on both the quality of life and daily functioning of older individuals, as well as their caregivers [4,5].

In comparison to the general population, elderly individuals are at greater risk of falls, especially falls that result in serious injury or death. Up to 39% of community-dwelling elderly people experience at least one fall per year [6]. The main risk factors for falls include balance and gait disorders, a history of falls, polypharmacy, advanced age, female gender, visual and cognitive impairments, and environmental factors such as home conditions and proper footwear [7]. People with cognitive decline are nearly eight times more likely to fall [7].

Studies in non-hospitalized geriatric persons have shown that NPS is associated with an increased risk of falls, decreased functional status, and lower quality of life in older individuals [8,9,10]. However, there are few studies evaluating the relationship between the cumulative number of NPS and the risk of falls among hospitalized geriatric individuals [11]. It is important to investigate this issue among hospitalized geriatric persons to key differences between the two populations. Hospitalized patients face increased medical complexity, unique environmental factors, medication side effects, and deconditioning from limited mobility. Additionally, they may experience more severe consequences from falls.

We examined the total number of caregiver-identified NPS among elderly people admitted to a hospital geriatric ward and their association with the risk of falls taking into account sex differences.

## 2. Materials and Methods

### 2.1. Design of Study, Participants, and Measurements

A cross-sectional study of all consecutive patients (*n* = 234) admitted to the geriatric ward at Leszek Giec Upper-Silesian Medical Centre of the Silesian Medical University in Katowice, Poland between January 2019, and January 2020, where a Comprehensive Geriatric Assessment (CGA) was completed. Our patient profile includes both acute and non-acute admissions. Reasons for admission included but are not limited to acute diseases or exacerbation of chronic conditions, falls and fractures, polypharmacy management, cognitive decline and dementia, functional decline, malnutrition and dehydration, pressure sores and wound care, and psychosocial issues. The CGA included patient history, physical examination, functional assessment, and ambulatory status. Data on the Barthel Index of Activities of Daily Living (Barthel Index—a 10-item scale measuring functional independence in ADLs, with scores ranging from 0—dependent, to 100—independent.) was used to determine functional status. The Mini-Mental State Examination (MMSE) [12], a 30-point screening tool, was used to assess global cognitive performance. The Berg Balance Scale, a 14-item test assessing balance and fall risk in older adults, was used to assess the risk of falls. Increased fall risk was defined by Berg scores of ≤40 [13]. The Barthel Index was used to evaluate the Activities of Daily Living and IADL for Instrumental Activities of Daily Living. Barthel Index scores range from 0 to 100, and MMSE scores range from 0 to 30; higher scores indicate better functional status. Patient histories were analyzed for basic data such as ambulatory status. Dementia was diagnosed according to recommendations from the National Institute on Aging–Alzheimer’s Association. The Charlson comorbidity index (CCI), a weighted score predicting 1-year mortality risk based on the presence of 19 comorbid conditions, was used to cumulatively quantify the average morbidity of our cohort.

### 2.2. The Neuropsychiatric Inventory–Questionnaire (NPIQ) 

Patient and patient–caregiver interviews, as well as a patient–caregiver Neuropsychiatric Inventory Questionnaire, were completed. The Neuropsychiatric Inventory–Questionnaire (NPIQ) was developed and cross-validated with the standard Neuropsychiatric Inventory (NPI) to provide a brief assessment of neuropsychiatric symptomatology in routine clinical practice settings. The NPIQ is adapted from the NPI, a validated informant-based interview that assesses NPS over the previous month [14]. The NPIQ includes 12 neuropsychiatric domains: delusions, hallucinations, agitation/aggression, dysphoria/depression, anxiety, euphoria/elation, apathy/indifference, disinhibition, irritability/lability, aberrant motor, nighttime disturbances, and appetite/eating disturbances. A caregiver distress scale is included in the NPIQ for assessment of the psychological impact of patient NPS on the caregiver. The NPIQ is designed to be a self-administered questionnaire completed by caregivers about patients for whom they provide care. Each of the 12 NPIQ domains contains a survey question that reflects the cardinal symptoms of that domain. Initial responses to each domain question are “Yes” (present in the last month) or “No” (absent in the last month). If the response to the domain question is “No”, the informant goes to the next question. If “Yes”, the informant then rates both the severity of the patient’s symptoms present within the last month on a 4-point scale (from 0 to 3) and the associated impact of the symptom manifestations on them (i.e., Caregiver Distress) using a 6-point scale (from 0 to 5). The NPIQ provides symptom severity and distress ratings for each symptom reported, and total severity and distress scores reflect the sum of individual domain scores. The Polish version of the NPIQ was translated by the lead author (KW) and is available for use through the Mapi Research Trust. 

### 2.3. Statistical Analysis

We analyzed data using STATISTICA version 13 (TIBCO Software, Arlington, TX, USA). Quantitative variables were expressed as average values with standard deviation, while categorical variables were shown as percentages. We compared variables between individuals with and without dementia, or with and without high fall risk, using the χ2 test for categorical variables and the nonparametric Mann–Whitney U test or t-student test for normal distribution quantitative variables. We verified normality using the Shapiro–Wilk test.

To determine an optimal cut-off point for predicting high fall risk based on the total number of neuropsychiatric symptoms and their intensity, we used receiver operating characteristic (ROC) curves. The accuracy of this prediction was defined by the area under the ROC curve (AUC). We used multivariate logistic regression to analyze the association between values equal to or greater than the cut-off point and high fall risk, with a *p*-value of less than 0.05 considered statistically significant.

We also used multivariate logistic regression to analyze the associations between high fall risk and the presence of particular neuropsychiatric symptoms.

### 2.4. Ethics

This study was conducted in accordance with the Declaration of Helsinki. The study protocol was registered with the Bioethical Committee of the Medical University of Silesia in Katowice, Poland. The project established that no procedure, including caregiver interview, exceeds standard procedures performed for patients hospitalized at the geriatrics department. The Bioethical Committee determined that in the context of law, the study is not a medical experiment and does not require assessment by the bioethical committee (Letter KNW/0022/KB1/196/18). Based on this decision, written informed consent from study participants was not required for our study, nor was separate patient consent required for our statistical analysis or research since patient data is not disclosed outside internal hospital ward staff. This decision enabled us to include patients with dementia in the study who would otherwise be excluded.

## 3. Results

### 3.1. Population Characteristics and Prevalence of Neuropsychiatric Symptoms 

In this study, 146 participants (62.39%) had dementia, while 88 participants (37.61%) did not. The study group had a mean age of 80.7 ± 6.6 years, with people with dementia being significantly older (81.6 ± 6.6 years) than those without (79.1 ± 6.4), *p* = 0.001. Women accounted for the majority (62.8%) of the study population.

The average number of neuropsychiatric symptoms (NPS) was 4.41 ± 3.09, with people with dementia experiencing a significantly higher number of NPS (5.25 ± 3.04) than those without dementia (3.02 ± 2.65), *p* < 0.001. Apathy was the most common neuropsychiatric disorder in the study group (58.1%) and among people with dementia (67.8%), while appetite/eating disturbances were the most common NPS among patients without dementia (46.6%) (see Table 1). Differences between men and women in individual NPS prevalence and NPS average intensity were not significant.

The prevalence of individual neuropsychiatric disorders is listed in Table 1. Furthermore, the Charlson Comorbidity Index was significantly higher in persons with dementia (*p* < 0.001, Table 1).

### 3.2. Dementia and Risk of Falls, Functional Status

The study revealed that individuals with dementia had a significantly higher risk of falls, with 80.60% of those with a Berg Balance Scale score of 40 points or less being at high risk, compared to 48.19% of those without dementia (*p* < 0.001). Furthermore, those with dementia had lower Barthel scores (*p* < 0.001) and lower ability to perform Instrumental Activities of Daily Living (IADL) (*p* < 0.001), as shown in Table 1.

Mean systolic and diastolic blood pressures were lower in the high fall risk group compared to the no high fall risk group (*p* = 0.014 and *p* = 0.023, respectively). In addition, individuals with an increased risk of falls were more likely to suffer from dementia (72.97%, *p* < 0.001), had a lower mean MMSE score (*p* < 0.001), and had lower ability to perform activities of daily living according to both the Barthel Index (*p* < 0.001) and the IADL scale (*p* < 0.001).

The high-risk of falls group had an average of 4.89 ± 3.02 NPS, which was significantly higher than the no high-risk of falls group’s average of 3.41 ± 3.09 (*p* < 0.001). Apathy was the most common NPS disorder in both risk fall groups, with a prevalence of 63.51% in the high-risk group and 49.28% in the no-high-risk group. Finally, the high-risk group had a lower probability of 10-year survival according to the Charlson Comorbidity Index (*p* < 0.001), as indicated in Table 1. 

### 3.3. Receiver Operating Characteristic (ROC) Curve Analysis

**Figure 1:**
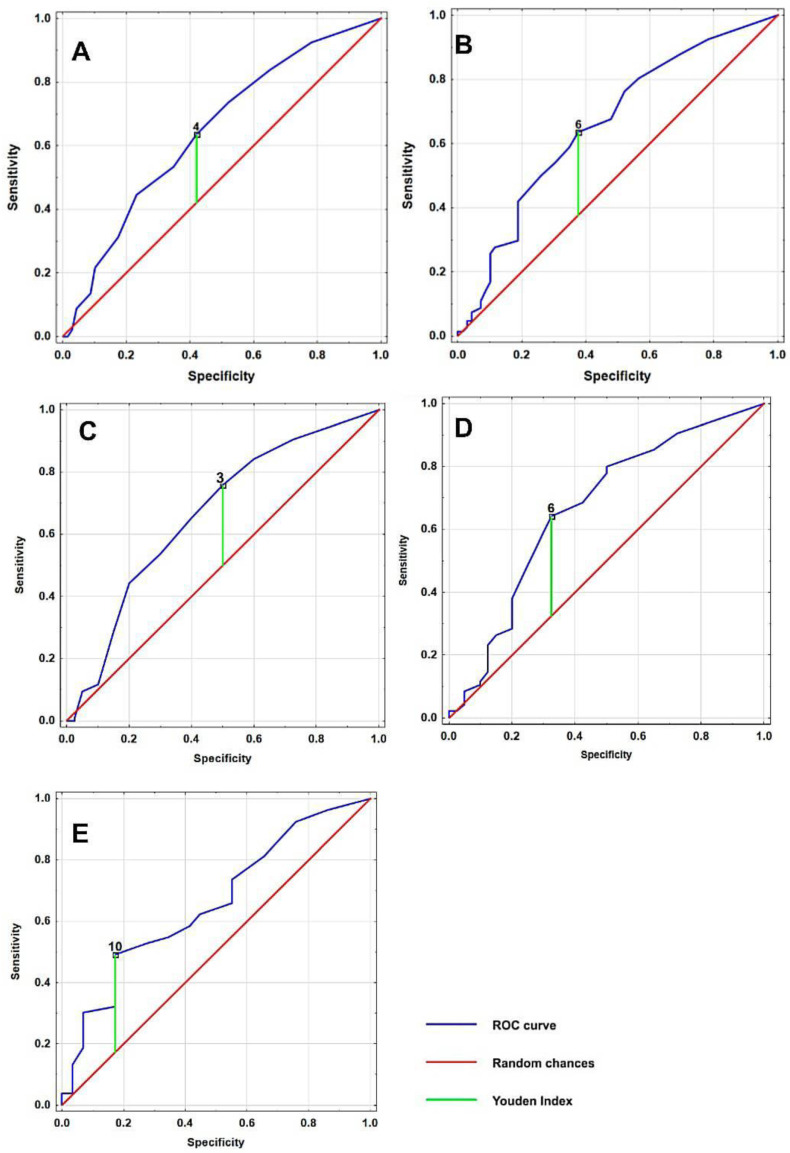
Receiver Operating Characteristic (ROC) curve analysis for the total number of neuropsychiatric symptoms ((**A**)—for the cohort, (**C**)—women only) and the total symptom intensity ((**B**)—for the cohort, (**D**)—women only, (**E**)—men only) in relation to a high fall risk in hospitalized geriatric individuals. The ROC curve analysis was used to assess the predictive accuracy of the total number of symptoms and their intensity for identifying high fall risk.

Analysis of the receiver operating characteristic (ROC) curves revealed significant cut-off points for the total number of NPS and their intensity in predicting high fall risk. The cut-off point for the total number of these symptoms was 4, while the cut-off for the total intensity of symptoms was 6, as shown in Table 2 and Figure 1. Analyzed by sex, for women, the cut-off point for total symptoms was 3, and for the total intensity of symptoms was 6. For men, the cut-off analysis of total symptoms was not significant, while the cut-off for total intensity of symptoms was 10 (Table 2, Figure 1).

To further investigate the relationship between NPS and fall risk, multivariate logistic regression was performed using the cut-off points in Table 2. Our data show a positive association between a high fall risk and having at least 4 NPS and, independently, the total intensity of symptoms of at least 6 (Table 3). In addition, high fall risk was associated with cut-off points for the total intensity of NPS for both men and women. Cut-off points were significant for association with high fall risk for the total number of NPS in women, but not in men (Table 3). 

### 3.4. Association between NPS and High Fall Risk

After adjusting for confounders, including age, sex, and individual neuropsychiatric syndromes, multivariate logistic regression analysis showed that the presence of hallucinations was associated with a high fall risk (OR = 6.54, 95%CI 1.43–30, *p* = 0.02).

**Figure 2:**
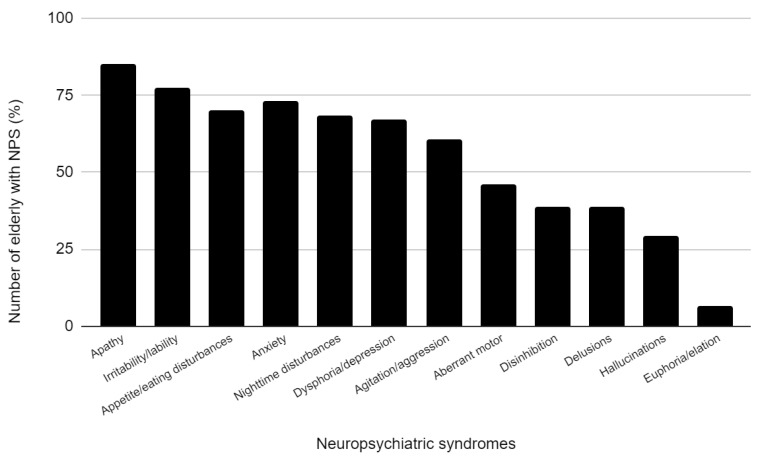
Frequency of neuropsychiatric symptoms in individuals at high fall risk (4 or more NPS).

Figure 2 presents the frequency of neuropsychiatric symptoms (NPS) in a group of individuals with a high fall risk (NPS 4 or more). The most common NPS was apathy, which was observed in 85.4% of patients, followed by irritability/lability in 77.37% of patients, and anxiety in 72.99% of patients.

## 4. Discussion

Among patients in our cohort, several neuropsychiatric symptoms were associated with an increased risk of falls. Other studies have examined the association of falls with individual NPS among geriatric persons. Most commonly, associations have been described between falls and hallucinations as well as delusions. Tapiainen in 2020 showed that hallucinations were associated with a risk of head injuries among persons with Alzheimer’s disease who were admitted to an acute care hospital after falling [15]. Isaacson showed in 2021 that persons with Parkinson’s disease and with delusions and hallucinations who were treated with antipsychotics were at increased risk of falls [16]. Association with falls has also been associated with other NPS, such as depression, among geriatric individuals diagnosed with joint disease [17]. A meta-analysis of 18 studies presented a significant association between increased risk of falls and anxiety [18]. The work of Holloway et al. described the association between anxiety disorders in older people and increased risk of falls, but only in men [19]. As such, there exists evidence that individual NPS may influence fall risk and the presence of NPS should alert the managing physician to possible fall interventions—especially among patients with hallucinations and or delusions. 

Several mechanisms may explain the relationship between hallucinations and falls. One possible explanation is that hallucinations may affect sensory perception, leading to disorientation and imbalance, which could increase the risk of falls. Visual hallucinations, in particular, could interfere with depth perception and spatial awareness, making it difficult for individuals to navigate their surroundings safely. Another possible mechanism is that hallucinations may cause distractions and confusion, leading to cognitive overload and an increased risk of falls. For example, individuals with auditory hallucinations may be distracted by voices and lose their balance as a result. Lastly, the medications used to treat hallucinations may have side effects that affect balance and coordination, increasing the risk of falls. Antipsychotic medications, in particular, have been associated with an increased risk of falls due to their sedative and anticholinergic effects [20].

We also found that there is an association between the cumulative total number of NPS and NPS intensity with fall risk in hospitalized geriatric patients. Surprisingly, this relatively easy-to-apply metric has been examined in just a few studies [8,9,21]. For example, Roitto et al. (2020) in Finland showed that NPS, especially NPS severity, may predict falls, and fall-related sequelae [8]. Fall prevention is a notoriously difficult problem to address among geriatric populations. A granular approach to individual NPS may lead to a better understanding of geriatric falls [22]. However, in the context of an institutional fall prevention program, simple metrics for assessing fall risk such as total NPS and total NPS intensity may be especially practical.

NPS among hospitalized geriatric individuals is often underdiagnosed [23,24]. Yet the identification of NPS is the basis for the implementation of hospital-based and home-based interventions. Our study shows that NPS is also common among geriatric inpatients without dementia, not just with dementia. According to several studies, NPS interventions have been shown to improve the quality of life among individuals with dementia. There are few studies that have examined NPS interventions among geriatric individuals with preserved cognitive function and NPS. For example, in 2020, Eikelboom et al. found that early and accurate identification of neuropsychiatric symptoms in persons with dementia may improve quality of life [25]. In a meta-analysis, Liang showed in 2022 that planned and professional physical activity has a positive effect on improving neuropsychiatric symptoms and the quality of life of individuals with Alzheimer’s disease [26]. Identifying neuropsychiatric symptoms in hospitalized patients is the first step in implementing appropriate interventions that may improve the quality of life of geriatric persons with NPS. Further studies are needed to understand the impact of NPS intervention in geriatric individuals without dementia.

We observed that fewer neuropsychiatric symptoms are associated with high fall risk in women compared to men. This sex-based difference could be attributed to several factors related to NPS prevalence and intensity differences between sexes. Reasons may be related to the finding that women might exhibit a broader range of neuropsychiatric symptoms, as suggested by other studies [27], which might dilute the association between specific symptoms and falls. In addition, certain neuropsychiatric symptoms such as apathy, which have been found to be more severe in men [28], might be more strongly associated with falls due to reduced motivation for engaging in physical activities, potentially increasing the risk of fall-related incidents. Lastly, it is essential to consider the potential influence of cultural and social factors, as men and women might have different healthcare-seeking behaviors, coping mechanisms, and social support networks that could impact the relationship between neuropsychiatric symptoms and falls [29]. Further research is needed to elucidate the underlying mechanisms contributing to this sex-based difference and to develop tailored interventions that address the unique risk factors for falls in both men and women.

A limitation of our study is that self-report and caregiver-reported measures of neuropsychiatric symptoms may not always be reliable or valid. Older adults may underreport their symptoms due to stigma or difficulty communicating, while caregivers may not have accurate information about the patient’s symptoms or may have their own biases. The Barthel Scale was developed for individuals with physical disabilities who may not be sensitive to other factors that can contribute to falls in older adults, such as cognitive or sensory impairments. While NPS were present in both patients with and without dementia, the impact of cognitive dysfunction alone on falls needs further exploration. Longitudinal studies that follow patients over time would be needed to establish causality and determine the direction of the relationship between neuropsychiatric symptoms and falls. Future research could build upon the current findings by increasing sample size and conducting longitudinal studies to establish causality and better understand the relationship between neuropsychiatric symptoms, IADL/ADL scores, and falls in elderly geriatric patients.

## 5. Conclusions

Neuropsychiatric symptoms among geriatric individuals present significant management challenges for clinicians. Our hospital-based geriatric ward cohort showed a positive association between an increased risk of falls as measured by the Berg scale, and both the total number of caregivers reported neuropsychiatric symptoms and their intensity. High fall risk was associated with fewer total NPS and fewer total NPS intensity in women than in men. In addition, we found that among neuropsychiatric symptoms, hallucinations were an independent risk factor for falling. These results suggest that fall prevention strategies should include the management of neuropsychiatric symptoms in hospitalized geriatric individuals.

## Figures and Tables

**Table 1:** Clinical characteristics of the cohort, including the distribution of patients with a high fall risk versus those without a high fall risk. Quantitative variables are reported as mean values with standard deviations (±SD), while categorical variables are presented as percentages with 95% confidence intervals.

Variable	Total Cohort	High Fall Risk	No High Fall Risk	*p*-Value	Women	Men	*p*-Value
	*n* = 234						
Age (years)	80.70 ± 6.60	81.99 ± 6.21	77.69 ± 6.03	<0.001	81.20 ± 6.21	79.95 ± 7.09	0.164
Sex (percentage of females)	63	64	58	0.379	-	-	-
Dementia (%)	62.40	72.97	37.68	<0.001	64.67	58.14	0.319
History of stroke (%)	11.50	17.93	4.35	0.007	10.96	15.12	0.355
Diabetes (%)	34.60	34.69	33.33	0.844	31.76	40.70	0.167
Systolic blood pressure (mmHg)	141. ± 24	138.2 ± 25	147 ± 22	0.014	145.01 ± 25	136 ± 22	0.005
Diastolic blood pressure (mmHg)	79.30 ± 12.8	77.92 ± 12.88	82.16 ± 12.81	0.023	81.21 ± 12.99	76.67 ± 11.69	0.005
Body mass index	26.70 ± 5.91	26.51 ± 5.25	27.20 ± 7.12	0.902	26.46 ± 5.29	27.21 ± 6.9	0.582
Barthel Index (score)	71.80 ± 27.30	61.59 ± 27.19	93.33 ± 7.16	<0.001	71.07 ± 25.90	71.45 ± 29.84	0.317
IADL Index (score)	17.55 ± 6.24	15.15 ± 5.26	22.90 ± 4.71	<0.001	17.58 ± 6.00	17.45 ± 6.63	0.913
Mini-Mental State Examination (score)	19.80 ± 8.31	17.97 ± 8.45	24.32 ± 5.3	<0.001	19.89 ± 8.13	19.74 ± 8.62	0.968
Total number of neuropsychiatric symptoms	4.41 ± 3.09	4.89 ± 3.02	3.41 ± 3.09	<0.001	4.41 ± 3.11	4.43 ± 3.13	0.947
Apathy/indifference, prevalence (%)	58.10	63.51	49.28	0.047	59.33	59.30	0.1
Apathy/indifference, intensity (score)	1.17 ± 1.17	1.31 ± 1.17	0.86 ± 1.07	0.008	1.20 ± 1.18	1.14 ± 1.14	0.722
Irritability/lability, prevalence (%)	53.80	57.43	44.93	0.085	52.00	55.81	0.572
Irritability/lability, intensity (score)	1.00 ± 1.07	1.07 ± 1.08	0.83 ± 1.04	0.10	0.97 ± 1.08	1.00 ± 1.08	0.618
Appetite/eating disturbances, prevalence (%)	53.80	55.40	47.83	0.298	54.00	51.16	0.67
Appetite/eating disturbances, intensity (score)	1.07 ± 1.14	1.14 ± 1.18	0.86 ± 1.03	0.105	1.09 ± 1.15	0.99 ± 1.13	0.512
Anxiety prevalence (%)	50.00	55.05	37.68	0.025	52.67	43.02	0.154
Anxiety intensity (score)	0.91 ± 1.05	1.00 ± 1.07	0.67 ± 0.98	0.002	0.98 ± 1.08	0.76 ± 0.96	0.133
Nighttime disturbances, prevalence (%)	47.90	54.05	37.68	0.025	45.33	52.33	0.301
Nighttime disturbances, intensity (score)	0.86 ± 1.04	0.99 ± 1.06	0.62 ± 0.91	0.013	0.81 ± 1.01	0.97 ± 1.07	0.257
Dysphoria/depression, prevalence (%)	44.40	44.59	40.58	0.58	45.33	41.86	0.605
Dysphoria/depression, intensity (score)	0.75 ± 0.99	0.78 ± 1.01	0.62 ± 0.91	0.345	0.83 ± 1.10	0.62 ± 0.84	0.255
Agitation/aggression, prevalence (%)	37.60	42.57	23.19	0.006	39.33	32.56	0.3
Agitation/aggression, intensity (score)	0.66 ± 0.96	0.78 ± 1.03	0.41 ± 0.81	0.006	0.68 ± 0.98	0.63 ± 0.98	0.491
Aberrant motor, prevalence (%)	29.10	35.13	18.84	0.015	30	30.40	0.822
Aberrant motor, intensity (score)	0.57 ± 0.99	0.68 ± 1.02	0.38 ± 0.84	0.02	0.59 ± 1	0.60 ± 1	0.851
Disinhibition, prevalence (%)	23.10	26.35	18.84	0.227	22.67	24.41	0.759
Disinhibition, intensity (score)	0.42 ± 0.86	0.51 ± 0.96	0.29 ± 0.67	0.16	0.42 ± 0.86	0.43 ± 0.86	0.814
Delusions, prevalence (%)	22.20	29.00	10.14	0.02	21.33	24.42	0.5
Delusions, intensity (score)	0.41 ± 0.83	0.55 ± 0.95	0.18 ± 0.6	0.002	0.40 ± 0.84	0.44 ± 0.86	0.62
Hallucinations, prevalence (%)	16.2	23.65	4.35	<0.001	14.67	22.09	0.147
Hallucinations, intensity (score)	0.29 ± 0.72	0.41 ± 0.8	0.09 ± 0.45	0.002	0.27 ± 0.71	0.35 ± 0.86	0.194
Euphoria/elation, prevalence (%)	4.70	2.70	7.24	0.231	4.67	4.65	0.1
Euphoria/elation, intensity (score)	0.09 ± 0.38	0.06 ± 0.31	0.11 ± 0.47	0.841	0.09 ± 0.40	0.08 ± 0.35	0.89
Number of medications	8.35 ± 4.05	8.36 ± 3.75	8.39 ± 4.77	0.787	8.45 ± 4.31	8.28 ± 3.57	0.868
Number of hospital days	5.96 ± 2.97	6.22 ± 3.13	5.53 ± 2.78	0.067	5.87 ± 2.90	6.12 ± 3.09	0.609
Charlson comorbidity index	10.80 ± 2.62	7.63 ± 2.03	6.39 ± 2.41	<0.001	7.03 ± 2.12	7.62 ± 2.41	0.04

**Table 2:** Optimal cut-off points for high fall risk.

Outcomes	Cut-Off Value	Sensitivity	Specificity	Youden Index	AUC	*p* Value
Complete cohort						
Total number of neuropsychiatric symptoms	4	0.64	0.42	0.21	0.65	<0.001
Intensity of total number of neuropsychiatric symptoms	6	0.64	0.38	0.26	0.66	<0.001
Women						
Total number of neuropsychiatric symptoms	3	0.79	0.5	0.26	0.67	0.002
Intensity of total number of neuropsychiatric symptoms	6	0.67	0.33	0.32	0.67	0.002
Men						
Total number of neuropsychiatric symptoms	6	0.51	0.26	0.18	0.65	0.062
Intensity of total number of neuropsychiatric symptoms	10	0.56			0.66	0.011

**Table 3:** Univariate logistic regression revealed associations between high fall risk and values greater than the ROC cut-off points. Odds ratio (OR), 95% confidence interval (95% CI).

Outcomes	OR	95%CI	*p* Value
Complete Cohort			
Total number of neuropsychiatric symptoms ≥ 4	2.50	1.35–4.65	0.004
Total neuropsychiatric symptom intensity ≥ 6	2.77	1.49–5.16	0.001
Women			
Total number of neuropsychiatric symptoms ≥ 3	3.90	1.69–8.98	0.001
Total neuropsychiatric symptom intensity ≥ 6	4.11	1.73–9.63	0.001
Men			
Total number of neuropsychiatric symptoms	(cutoffs not significant)		
Total neuropsychiatric symptom intensity ≥ 10	3.86	1.25–11.96	0.019

## Data Availability

Not applicable.

## References

[1] Aalten P., Verhey F.R., Boziki M., Bullock R., Byrne E.J., Camus V., Caputo M., Collins D., De Deyn P.P., Elina K. (2007). Neuropsychiatric syndromes in dementia. Results from the European Alzheimer Disease Consortium: Part I. Dement. Geriatr. Cogn. Disord..

[2] Fernández-Martínez M., Molano A., Castro J., Zarranz J.J. (2010). Prevalence of neuropsychiatric symptoms in mild cognitive impairment and Alzheimer’s disease, and its relationship with cognitive impairment. Curr. Alzheimer Res..

[3] Liew T.M. (2020). Neuropsychiatric symptoms in cognitively normal older persons, and the association with Alzheimer’s and non-Alzheimer’s dementia. Alzheimers Res. Ther..

[4] Majer R., Adeyi O., Bagoly Z., Simon V., Csiba L., Kardos L., Hortobágyi T., Frecska E. (2020). Neuropsychiatric symptoms, quality of life and caregivers’ burden in dementia. Open Med..

[5] Liampas I., Siokas V., Lyketsos C.G., Dardiotis E. (2022). The Relationship between Neuropsychiatric Symptoms and Cognitive Performance in Older Adults with Normal Cognition. Medicina.

[6] Moncada L.V.V., Mire L.G. (2017). Preventing Falls in Older Persons. Am. Fam. Physician.

[7] Rubenstein L.Z., Josephson K.R. (2002). The epidemiology of falls and syncope. Clin. Geriatr. Med..

[8] Roitto H.M., Öhman H., Salminen K., Kautiainen H., Laurila J., Pitkälä K.H. (2020). Neuropsychiatric Symptoms as Predictors of Falls in Long-Term Care Residents with Cognitive Impairment. J. Am. Med. Dir. Assoc..

[9] Roitto H.M., Kautiainen H., Öhman H., Savikko N., Strandberg T.E., Raivio M., Laakkonen M.L., Pitkälä K.H. (2018). Relationship of Neuropsychiatric Symptoms with Falls in Alzheimer’s Disease—Does Exercise Modify the Risk?. J. Am. Geriatr. Soc..

[10] Saari T., Hallikainen I., Hintsa T., Koivisto A.M. (2020). Neuropsychiatric symptoms and activities of daily living in Alzheimer’s disease: ALSOVA 5-year follow-up study. Int. Psychogeriatr..

[11] Mazur K., Wilczyński K., Szewieczek J. (2016). Geriatric falls in the context of a hospital fall prevention program: Delirium, low body mass index, and other risk factors. Clin. Interv. Aging.

[12] White N., Leurent B., Lord K., Scott S., Jones L., Sampson E.L. (2017). The management of behavioural and psychological symptoms of dementia in the acute general medical hospital: A longitudinal cohort study. Int. J. Geriatr. Psychiatry.

[13] Buhr G.T., White H.K. (2007). Difficult Behaviors in Long-term Care Patients with Dementia. J. Am. Med. Dir. Assoc..

[14] Kaufer D.I., Cummings J.L., Ketchel P., Smith V., MacMillan A., Shelley T., Lopez O.L., DeKosky S.T. (2000). Validation of the NPIQ, a Brief Clinical Form of the Neuropsychiatric Inventory. J. Neuropsychiatry Clin. Neurosci..

[15] Tapiainen V., Lavikainen P., Koponen M., Taipale H., Tanskanen A., Tiihonen J., Hartikainen S., Tolppanen A.M. (2020). The Risk of Head Injuries Associated with Antipsychotic Use Among Persons with Alzheimer’s disease. J. Am. Geriatr. Soc..

[16] Isaacson S.H., Citrome L. (2022). Hallucinations and delusions associated with Parkinson’s disease psychosis: Safety of current treatments and future directions. Expert Opin. Drug Saf..

[17] Byun M., Kim J., Kim M. (2020). Physical and Psychological Factors Affecting Falls in Older Patients with Arthritis. Int. J. Environ. Res. Public Health.

[18] Hallford D.J., Nicholson G., Sanders K., McCabe M.P. (2017). The Association Between Anxiety and Falls: A Meta-Analysis. J. Gerontol. B Psychol. Sci..

[19] Holloway K.L., Williams L.J., Brennan-Olsen S.L., Morse A.G., Kotowicz M.A., Nicholson G.C., Pasco J.A. (2016). Anxiety disorders and falls among older adults. J. Affect. Disord..

[20] Wilczyński K., Gorczyca M., Gołębiowska J., Szewieczek J. (2021). Anticholinergic Burden of Geriatric Ward Inpatients. Medicina.

[21] Sylliaas H., Selbaek G., Bergland A. (2012). Do behavioral disturbances predict falls among nursing home residents?. Aging Clin. Exp. Res..

[22] Balzer K., Bremer M., Schramm S., Lühmann D., Raspe H. (2012). Falls prevention for the elderly. GMS Health Technol. Assess..

[23] Gamble L.D., Matthews F.E., Jones I.R., Hillman A.E., Woods B., Macleod C.A., Martyr A., Collins R., Pentecost C., Rusted J.M. (2022). Characteristics of people living with undiagnosed dementia: Findings from the CFAS Wales study. BMC Geriatr..

[24] Hodgson N.A., Gitlin L.N., Winter L., Czekanski K. (2011). Undiagnosed illness and neuropsychiatric behaviors in community residing older adults with dementia. Alzheimer Dis. Assoc. Disord..

[25] Eikelboom W.S., Singleton E., van den Berg E., Coesmans M., Mattace Raso F., van Bruchem R.L., Goudzwaard J.A., de Jong F.J., Koopmanschap M., den Heijer T. (2019). Early recognition and treatment of neuropsychiatric symptoms to improve quality of life in early Alzheimer’s disease: Protocol of the BEAT-IT study. Alzheimers Res. Ther..

[26] Liang Y.J., Su Q.W., Sheng Z.R., Weng Q.Y., Niu Y.F., Zhou H.D., Liu C.B. (2022). Effectiveness of Physical Activity Interventions on Cognition, Neuropsychiatric Symptoms, and Quality of Life of Alzheimer’s Disease: An Update of a Systematic Review and Meta-Analysis. Front. Aging Neurosci..

[27] Tao Y., Peters M.E., Drye L.T., Devanand D.P., Mintzer J.E., Pollock B.G., Porsteinsson A.P., Rosenberg P.B., Schneider L.S., Shade D.M. (2018). Sex Differences in the Neuropsychiatric Symptoms of Patients with Alzheimer’s Disease. Am. J. Alzheimer’s Dis. Dement..

[28] Eikelboom W.S., Pan M., Ossenkoppele R., Coesmans M., Gatchel J.R., Ismail Z., Lanctôt K.L., Fischer C.E., Mortby M.E., van den Berg E. (2022). Sex differences in neuropsychiatric symptoms in Alzheimer’s disease dementia: A meta-analysis. Alzheimer’s Res. Ther..

[29] Thompson A.E., Anisimowicz Y., Miedema B., Hogg W., Wodchis W.P., Aubrey-Bassler K. (2016). The influence of gender and other patient characteristics on health care-seeking behaviour: A QUALICOPC study. BMC Fam. Pract..

